# Design and thermal imidization of new 1,3-thiazine-based polyimides and copolyimides for high-performance corrosion inhibition

**DOI:** 10.1038/s41598-025-22235-4

**Published:** 2025-10-27

**Authors:** Marwa M. Sayed, Shimaa M. Ahmed, Mohamed Abdel-Hakim, El-Refaie Kenawy, Kamal I. Aly

**Affiliations:** 1 Chemistry Department, Faculty of Science, New Valley University, El-Kharja, 72511 Egypt; 2 Polymer Research Laboratory 122, Chemistry Department, Faculty of Science, Assiut University, Assiut, 71516 Egypt; 3 Chemistry Department, Faculty of Science, Al-Azhar University, Assiut, 71524 Egypt; 4 Polymer Research Group, Chemistry Department, Faculty of Science, Tanta University, Tanta, 31527 Egypt

**Keywords:** Polyimides, Co-polyimides, Coating, Anti-corrosion, Thermal imidization, Polythiazine., Chemistry, Materials science

## Abstract

A new polyimide, poly(1,3-thiazine imide) (PTzI), together with two co-polyimides (CoPTz-DsI and CoPTz-HaI), were synthesized from the monomer named 4,4’-(1,4-phenylene)bis(6-(benzene-2-yl)-6 H-1,3-thiazine-2-amine) (PTA), which has a di-1,3-thiazine heterocyclic structure. The structure of PTA was confirmed with FT-IR, along with NMR spectra and mass spectroscopy. A thermal imidization process was used to prepare PTzI from the corresponding polyamic acid (PAA), which was obtained by polycondensation of PTA with 3,3′,4,4′-benzophenone tetracarboxylic dianhydride (BTDA). Similarly, the copolymers were synthesized via thermal imidization of PAAs derived from the polycondensation of PTA with 4,4′-diaminodiphenyl sulfone (DDS) or 1,6-hexane diamine (HAD), in addition to BTDA. The structure and imidization of each polymer were verified at various curing temperatures using Fourier transform infrared FT-IR spectroscopy. X-ray diffraction verified their amorphous nature, while scanning electron microscopy (SEM) morphological investigation indicated different levels of homogeneity and compactness. The rigid aromatic backbones and imide linkages provide exceptional thermal stability and chemical resistance, demonstrating their high performance. Thermogravimetric study (TGA and derivative TGA) showed that the materials had outstanding thermal stability, with high degradation initiation and initial decomposition temperatures (Td_5%_) in the range of 294–418 °C, and char yields of 52–63% at 800 °C. In acidic environments, the synthesized polymers have shown exceptional corrosion prevention performance for mild steel MS, according to electrochemical evaluations. Due to its structural composition rich in 1,3-thiazine structure, PTzI exhibited the highest inhibition efficiency (99.4%). Additionally, CoPTz-HaI and CoPTz-DsI demonstrated excellent efficiencies of 95.2% and 96.4%, respectively. The inhibitory performance was shown to be improved as the PTA concentration increased, suggesting that it may be used to create more sophisticated coatings that resist corrosion.

**Supplementary Information:**

The online version contains supplementary material available at 10.1038/s41598-025-22235-4.

## Introduction

The exceptional mechanical strength, resistance to solvents, and thermal stability of polyimide (PI) make it an outstanding performance polymer. A wide variety of advanced technology applications, including those in the aerospace sector, medical and electrical devices, sensors, film, fiber, nanofiber, membrane, foam, adhesive, and coating, utilize PIs due to their exceptional properties^1–4^. PIs have numerous applications, including thermal control coatings and protective layers for electronic devices and space applications^5^. These PIs have excellent optical properties, such as low solar absorption and infrared emission, radiation resistance, low density, toughness, flexibility, and high mechanical stability^6,7^. The materials exhibit a broad service temperature, possess enhanced electrical properties, and demonstrate a dielectric constant between 3.4 and 3.5^8^. In PIs, the functional imide link (–CO–N–CO–) is often incorporated as a five-membered cyclic structure. Linear imide bonds are potentially possible, but they are unstable and hard to make, so they can’t be used in high-performance polymers. In contrast, cyclic imide structures, particularly those based on aromatic backbones, provide rigidity and strong intermolecular interactions, which are responsible for the exceptional thermal stability, mechanical strength, and chemical resistance of polyimide^9^. In PI synthesis, there are two primary methods for introducing imide groups: (i) the direct polymerization or polycondensation of monomers that already contain imide units, and (ii) the polycondensation of dianhydrides with diamines to form polyamic acid (PAA) intermediates, which are subsequently cyclodehydrated into imide rings during polymerization^10^. The most difficult part of designing a polyimide molecular structure is achieving a balance between the PI’s dielectric, mechanical, thermal, and molecular weight characteristics. For instance, by including rigid structures like benzene rings, benzimidazoles, benzoxazoles, or benzothiazole rings in PIs, their thermal stability may be enhanced. The increased stiffness, however, reduces their solubilities and makes subsequent processing difficult. Adding aliphatic chains and ether bonds to PI structures improves their processability and solubility, but it decreases their thermal stability. Sometimes these characteristics are at variance with one another, and it’s important to maintain a balance between them for certain uses^11^. Aromatic PIs often have a lower coefficient of thermal expansion and better heat persistence, but they are opaque due to their powerful charge transfer complex. While aromatic PIs have better thermal performance, certain aliphatic PIs have better transparency^12,13^.

Corrosion does a lot of damage to society because it breaks down new building materials and structures. The continuous corrosive degradation of materials destroys multiple material qualities. The weakness of systems and the resulting undesirable appearance are indications of corrosion^14,15^. It can be found in nearly every substance when they are in their respective operational settings. Furthermore, the expenses of dealing with corrosion are quite costly, with an expenditure^16^. Protecting against corrosion has emerged as a critical concern in addressing this issue. Different methods for protecting metals from corrosion have emerged during the last few decades. These include cathodic protection, anodic passivation, coatings on the surface (organic, inorganic, and organic-inorganic hybrid coatings), and immersion treatment with corrosion inhibitors^11,17,18^. When it comes to protecting metals against corrosion, organic coatings have long been considered the gold standard. Researchers are interested in using organic molecules with π-electrons in triple or conjugated double bonds and heteroatoms, such as nitrogen, oxygen, phosphorus, and sulfur, as inhibitors. Adsorption of organic substances forms a coating on metal surfaces, preventing corrosion. A compound’s inhibitory efficacy depends on its adsorption ability, molecular characteristics, molecular planarity, and the interaction between its p-orbital and iron’s d-orbital. Therefore, organic compounds that can donate electrons to the metal surface’s vacant d-orbital and take free electrons via their anti-bonding orbitals are effective corrosion inhibitors^19–23^. Thiazine and its derivatives have been identified as potential organic inhibitors due to their thiazine nucleus, which includes a six-ring system with two heteroatoms, nitrogen and sulfur, positioned at the 1 and 3 locations of the ring, making them advantageous for corrosion protection^24,25^.

The use of PI as a coating to impart the industrial equivalent performance is an intriguing but challenging prospect. Despite its elevated melting point and poor solubility, PI has exceptional mechanical characteristics due to its stiff aromatic ring architecture and significant intermolecular forces^26^. Coating the substrate directly with PI via a melt or solution technique becomes challenging due to this feature. The coating method typically begins with a soluble polyamic acid (PAA) precursor and ends with the matching PI, which is generated by the high-temperature thermal imidization procedure (250–300 °C)^27–29^. The remarkable features of polyimide (PI) coatings, such as their remarkable robustness to both high as well as low temperatures, strong barrier qualities, and great adherence to metal surfaces, make them ideal for maintaining metals in corrosive environments^30–32^.

In this study, new PI and co-polyimides CPIs with a thiazine ring in the main chains are synthesized and characterized. Our objective is to increase the number of hetero atoms containing nitrogen and sulfur to suppress corrosion employing a heterocyclic ring. The diamine thiazine monomer was synthesized via the incorporation of thiourea into the chalcone molecule. PI and CPIs are prepared by thermal imidization from thiazine monomer PTA, 4, 4’-diamino diphenyl sulphone DDS, or 1,6-diamino hexane HAD with 3, 3’, 4, 4’-benzophenone tetracarboxylic dihydride (BTDA). Their structures were confirmed, compared, and their effectiveness as corrosion inhibitors was assessed, demonstrating enhanced inhibition.

## Experimental

### Materials

The following materials were obtained from Sigma Aldrich: terephthaldehyde, 1,6-hexane diamine (HAD), sulfuric acid (H_2_SO_4_ 98%), and ethanol absolute (EtOH), which were used completely as supplied. Diamethylacetamide (DMAc), dimethyl formamide (DMF), dimethyl sulfoxide (DMSO), dichloromethane (DCM), and acetophenone, all of which are anhydrous and are from Alfa Chemicals. 3, 3’, 4, 4’-benzophenone tetracarboxylic dianhydride (BTDA) and 4,4’-diamino diphenyl sulfone (DDS) from (Merck). Thiourea, sodium carbonate, acetone, toluene, hydrochloric acid (HCl), sodium hydroxide NaOH, potassium hydroxide KOH, sodium bicarbonate (NaHCO_3_), and magnesium sulfate (El Nasser chemicals) are used without undergoing any kind of purifying procedure.

### Measurements

The measurement of Fourier transform infrared spectroscopy (FT-IR) is conducted utilizing the KBr technique with Shimadzu 2110 PC scanning spectrometers. Nuclear magnetic resonance (^1^H-NMR) spectra were obtained utilizing deuterated DMSO as the solvent, employing a JEOL (JNM-ECZ 500R/S1) spectrometer. Gas Chromatography-Mass Spectrometry (GC-MS) spectra were acquired using a Hewlett-Packard 5973 MSD spectrometer, employing EI (70 eV), in conjunction with a Hewlett-Packard Agilent 6890 chromatograph, which was fitted with an HP-5 MS column (30 m x 0.25 mm x 0.25 μm). X-ray diffraction (XRD) was conducted using a Philips X-ray PW 1710 diffractometer with Ni-filtered CuKα radiation. With a heating rate of 10 °C per minute, the TA Q-600 Thermal Analyzer measures thermogravimetric analysis (TGA) in a nitrogen environment (N_2_). Differential scanning calorimeter (DSC) analyses were conducted utilizing a TA Q-20 in N_2_ atmosphere. To determine the surface morphology of the polymers, the coating technique was observed using a scanning electron microscope (SEM) from Hitachi, model S-4800. The EG&G Potentiostat/Galvanostat Model 273 A and standard corrosion measuring methods 352/252 are used in the electrochemical studies conducted at the IBM Program (Al-Azhar University - Asyut). Open circuit potential and polarity tests (linear, Tafel plot) are included in these evaluations.

### Synthesis of 3, 3’-(1,4-phenylene) Bis (1- phenyl prop-2-en-1-one) (bis chalcone) BCO

A combination of acetophenone (2.4 g, 0.02 mol) and terephthaldehyde (1.34 g, 0.01 mol) in about 30 mL of absolute ethanol and drops of KOH (30%) was stirred overnight at the ambient temperature in a flask equipped with a condenser. For 24 h, the solution was refrigerated before being poured into ice-cold water and acidified with HCl. After separating the precipitate, it was dissolved in DCM, and then it was rinsed with saturated NaHCO_3_ and water. The compound yield was 82%; it was recrystallized from ethanol as a canary powder with a melting point (m.p.) of 175 °C. ^1^H-NMR (500 MHz, DMSO-d_6_, δ, ppm) at ẟ: 8.16 (m, 1H, =CH_a_); 7.76 (d, 1H, =CH_b_); 7.54–7.98 (m, Ar-H). ^13^C-NMR at ẟ: 189.69 ppm for (C = O), 143.62 ppm for (C_a_=C-C = O), 123.60 ppm for (C = C_b_-C = O), (129.13, 129.35, 129.95, 133.78, 137.22, 138.04 ppm) for aromatic carbons. The molecular ion peak at m/z = 338.2 was observed in the mass spectrum, which is consistent with its molecular formula (C_24_H_18_O_2_).

### Synthesis of 4,4’-(1,4-phenylene)bis(6-(benzene-2-yl)-6 H-1,3-thiazine-2- amine) PTA monomer

In a combination of (3.38 g, 0.01 mol) of chalcone and (1.52 g, 0.02 mol) of thiourea in 30 mL of ethanolic NaOH, the constituents were stirred constantly for three hours. Following an hour of vigorous stirring, 500 mL of cold water was added to the reaction. The mixture was allowed to cool for another 24 h. The resultant precipitate underwent filtration, washing, and recrystallization. The compound yield is 75% with (m.p.) 315 °C. ^1^H-NMR (500 MHz, DMSO-d_6_, δ, ppm) at ẟ: 8.4 (s, 2H_c_, NH_2_ ), 7.22 (d,1H, =CH_a_), 4.3 (s, 1H_b_, -CH_b_-CH=); 7.44–7.96 (m, Ar-H). ^13^C-NMR at ẟ: 160 − 159 for (C = N, C-NH_2_), 34.5 for (CH_b_-S), and 127–129 for (carbon of benzene rings). The molecular ion peak at m/z = 454.6 was observed in the mass spectrum, which is consistent with its molecular formula (C_26_H_22_N_4_S_2_).

### General synthesis of polyimide

A diamine compound (0.001 mol) was placed into a 50 mL flask, followed by the addition of 15 mL of solvent DMAc, and stirred until fully dissolved. Subsequently, (0.001 mol) of BTDA was introduced into the mixture under a nitrogen atmosphere. The viscous PAA solution was obtained by stirring the reaction solution at room temperature for 24 h. The PAA solution was poured onto a clean plate and subsequently placed in the oven to dry the solvent. The imidization process involves curing in an oven following a heating procedure of 150℃ for 2 h, 200℃ for 2 h, and 300℃ for 2 h to achieve polyimide.

### Synthesis of poly 1,3-thiazine imide (PTzI)

A 50 mL flask containing 0.001 mol (0.455 g) of PTA in 15 mL of DMAc was stirred until fully dissolved, after which about 0.001 mol (0.322 g) of BTDA was added and stirred for 24 h. The viscous PAA solution is evaporated and then polymerized using thermal imidization. The polyimide is produced by curing in an oven at a temperature of 150℃ for 2 h, 200℃ for 2 h, and 300℃ for 2 h.

### Synthesis of copoly 1,3-thiazine and 4,4’-diamino diphenyl sulfone (DDS) imide (CoPTz-DsI)

The copolymer (CoPTz-DsI) is synthesized in the same way as the polymer: in a flask, dissolve 0.0005 mol (0.228 g) of PTA and 0.0005 mol (0.124 g) of DDS in 15 mL of DMAc by stirring until completely combined. Then, add about 0.001 mol (0.322 g) of BTDA and stir for 24 h. Thermal imidization is used to polymerize the viscous PAA solution after it has been evaporated. The co-polyimide is made by curing it in an oven at 150 °C for two hours, 200 °C for two hours, and 300 °C for two hours.

### Synthesis of copoly 1,3-thiazine and 1,6-hexane diamine (HaD) imide (CoPTz-HaI)

The copolymer (CoPTz-HaI) is prepared by dissolving about 0.0005 mol (0.228 g) of PTA and 0.0005 mol (0.058 g) of HAD in 15 mL of DMAc in a flask, stirring until it is completely dissolved. Subsequently, include about 0.001 mol (0.322 g) of BTDA and agitate for 24 h, followed by thermal imidization of the resultant PAA as mentioned previously.

### Surface and medium preparation for study

Samples of mild steel (MS) have an iron (Fe) content of more than 98% by weight. To execute electrochemical investigations, MS samples were divided into pieces of 1 × 1 × 1 cm³. After being degreased with acetone, the surfaces of all the samples that were evaluated were polished using sandpapers of varying thicknesses (1200 and 1400 grit), and finally, they were allowed to dry.

### Solution preparation for inhibitors and corrosives

As for the synthesis of PIs, the inhibitors (PIs and co-polyimides) are typically synthesized using the same procedure as in the more analogous setting of the above experiments. For the PTzI tests, 0.2 mg of PTA and 0.1 mg of BTDA are used. For the PTz-DsI or PTz-HaI tests, 0.3 mg of PTA and (0.37 mg of DDS or 0.2 mg of HAD) with 0.1 mg of BTDA, respectively, are mixed in 3000 µL of DMAc. As a result of vigorous stirring for 24 h, thick PAAs were produced. Once the MS plate (1 × 1 × 1 cm³) had been cleaned, the produced PAAs were cast. Furthermore, the co-polyimides and PI were formed by curing the coating at 300 °C for 24 h. According to the micrometer caliper, the substance’s thickness on the MS electrode was approximately 5.5 μm. The corrosive acid solution was made by diluting an analytical 98% H_2_SO_4_ solution with high-purity distilled water. The corrosive material has to be submerged for the open-circuit voltage to work.

## Results and discussion

**Fig. 1:**
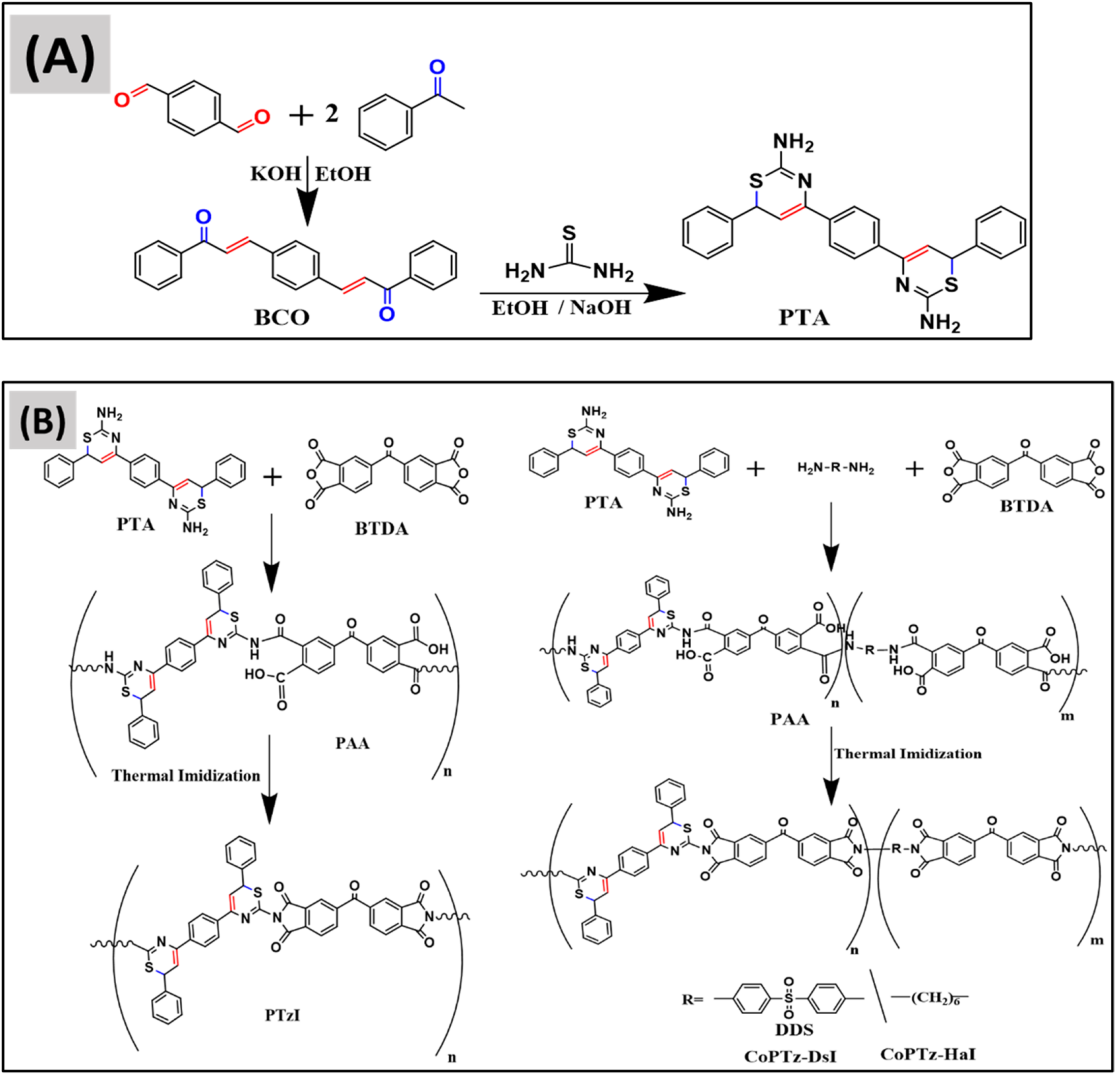
**(A)** Synthesis of bischalcon BCO and diamino dithiazine PTA monomer molecule. (**B)** Synthesis of PAA and PTzI, CoPTz-DsI, and CoPTz-HaI through thermal imidization.

**Fig. 2:**
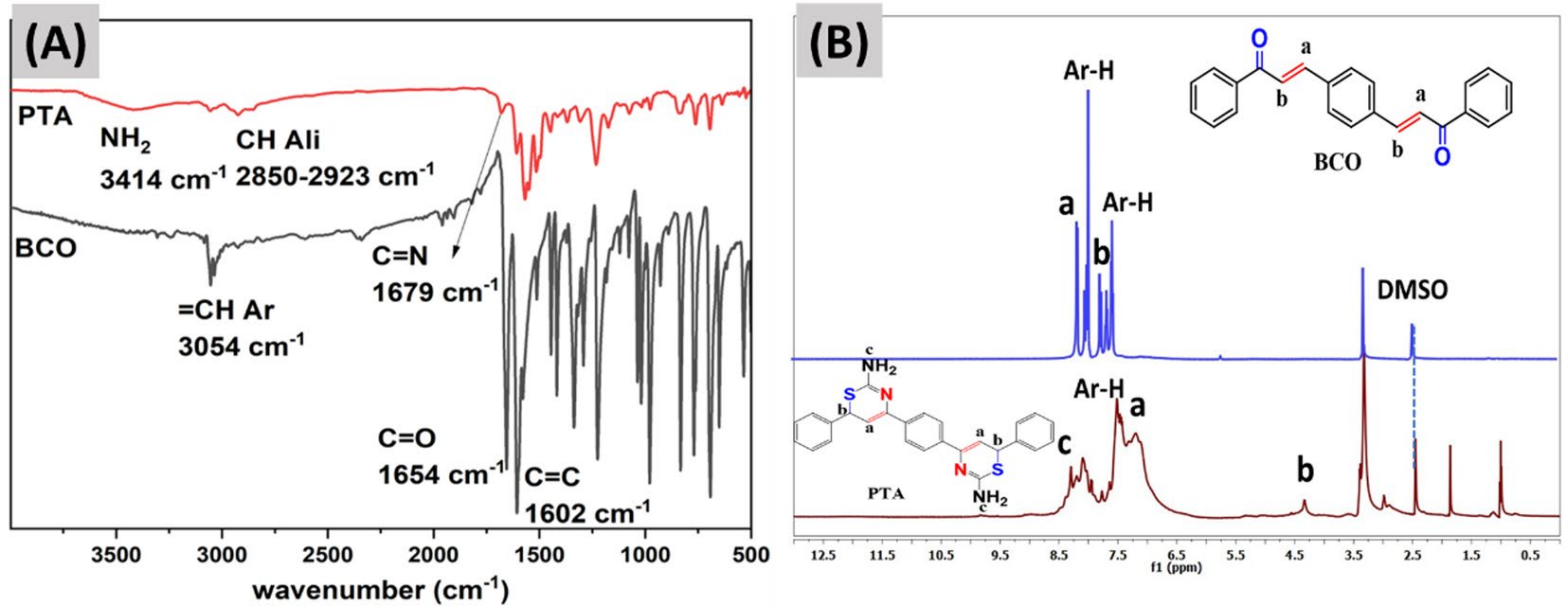
(**A**) FT-IR. (**B**) ^1^H-NMR of BCO and PTA compounds.

The monomer PTA was synthesized via a two-step condensation reaction^33^ as outlined in Fig. 1A. The initial stage in the synthesis of chalcone compound BCO is the aldol condensation of terephthaldhyde with acetophenone in alcoholic KOH^34,35^. The subsequent step entails the straightforward condensation of the BCO with thiourea in a basic medium, resulting in the production of the diamino dithiazine compound PTA with a favorable yield. The structure of each compound has been verified through FT-IR and NMR spectroscopy analysis. Figure 2A displays the FT-IR of BCO and PTA; the carbonyl C = O group of BCO is observed at 1654 cm^− 1^, while the olefinic double bond C = C appears at 1602 cm^− 1^, which is attributed to the conjugation of these two groups^36,37^. The aromatic HC = C stretching band is observed at 3054 cm^− 1^, while the C = C aromatic band is observed at 1445 cm^− 1^. The FT-IR analysis of PTA reveals the absence of the C = O bond and the emergence of the C = N bond at 1679 cm^− 1^, attributed to conjugation with the C = C bond. Additionally, the NH_2_ group is observed at 3400 cm^− 1^, accompanied by a pronounced bending mode at 1568 cm^− 1^. Aromatic CH appears at 3055 cm^− 1^, whereas aliphatic CH is at 2923 and 2850 cm^− 1^. The C = C conjugated diene is observed at 1607 cm^− 1^, while the aromatic C = C double bond is observed at 1447 cm^− 1^. The ^1^H-NMR spectra of BCO and PTA in DMSO-d_6_ are presented in Fig. 2B. The observed BCO signals are at δ ppm 7.56 (s, 1H) for (H_b_-C = CO), 7.16–7.92 for (H-Ar of aromatic), and 8.15 for (C = H_a_C-Ar). The noticed signals for PTA δ ppm are at 8.3 (s, 2 H, NH_2_), 4.5 (s, 1H, CH_a_), 6.5 (s, 1H, CH_b_=), and within the range of 6.8–7.7 for (Ar-H protons). The noticed signals for PTA δ ppm are at 8.4 (s, 2H_c_, NH_2_), 4.3 (s, 1H, CH_b_), 7.22 (s, 1H, CH_a_=), and within the range of 7.44–7.96 for (Ar-H protons). Figure S1 presents the ^13^C-NMR spectra of BCO and PTA in DMSO-d_6_, with BCO δ ppm values of 186.85 for (C = O), 144.5 for (C_a_H=CH), 123.15 for (CH = C_b_H), and 130-138.05 for (Ar-Carbons). Signals for PTA were seen at 160 − 159 for (C = N, C-NH_2_), 34.5 for (CH_b_-S), and 127–129 for (carbon of benzene rings). The molecular ion peak for BCO is noticed at m/z 338.2, and for PTA is observed at m/z 454.6 in mass spectrometry Figure S2, corresponding to its molecular weight. The results presented above demonstrate the effectiveness of the monomer preparation process.

The polyimide and co-polyimides are synthesized by reacting diamine monomers with BTDA in DMAc at ambient temperature under nitrogen, resulting in a PAA solution that gradually increases in viscosity over time. The resulting solution undergoes thermal imidization at varying curing temperatures for fixed durations to produce the final polyimide for coating applications^38,39^. Figure 1B illustrates the synthesis of polyimide by the reaction of equimolar quantities of PTA and BTDA in DMAc solvent, followed by heat curing of the resultant combination to get the desired polyimide. The synthesis of co-polyimides is shown in Fig. 1B, using the reaction of equimolar quantities of different diamines, either (PTA with DDS) or (PTA with HAD), with a stoichiometric amount of BTDA equivalent to the total moles of the diamines.

**Fig. 3:**
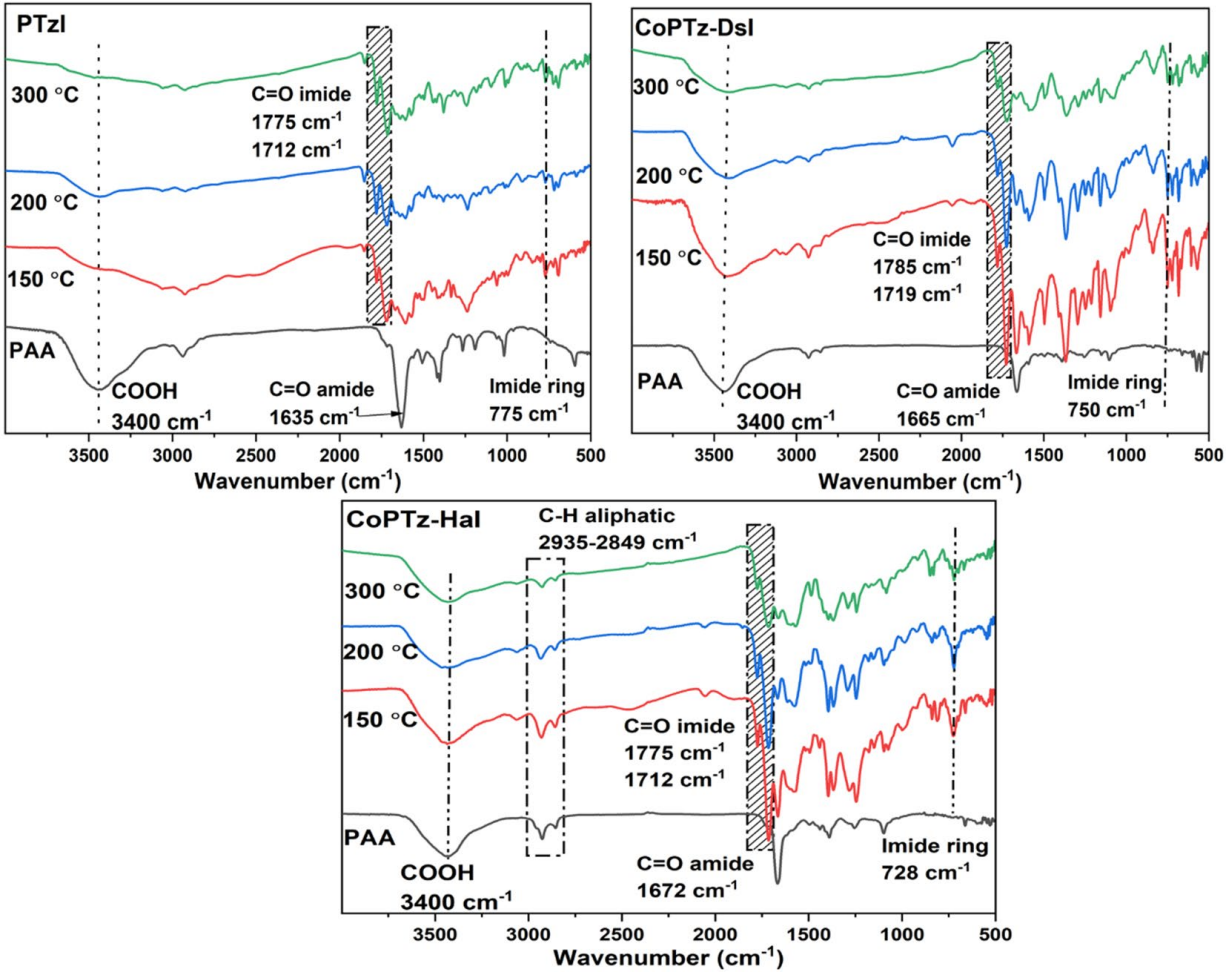
FT-IR of PAA and cured PtzI, CoPTz-DsI, and CoPTz-HaI at different curing temperatures, 150 °C, 200 °C, and 300 °C.

The resultant polymer samples were subjected to FT-IR measurements at various imidization temperatures to determine when the imidization process was completed and the formation of polyimide. The FT-IR spectra of PtzI, CoPTz-DsI, and CoPTz-HaI are shown in Fig. 3, after being cured at 150 °C, 200 °C, and 300 °C for 2 h. The intensity of the absorption peaks of functional groups of PAA decreased during curing, and all peaks of PI showed an increase as the temperature rose. This indicates that the conversion of the PAA solution into PI is proceeding, with complete imidization achieved at a temperature higher than 200 °C. The typical C = O peaks of the amide group in PAA were observed at 1635 cm^− 1^ for PTzI, 1665 cm^− 1^ for CoPTz-DsI, and 1672 cm^− 1^ for CoPTz-HaI. For all PAA of the samples, the symmetric carboxylate COO^−^ ion stretch bands are at 1405 cm^− 1,^ and the C = O carbonyl stretch from carboxylic acid is at 1720 cm^− 1^. The bands distinctive of the amide bond decrease as the reaction and curing process progress, and the C = O stretch imide peaks arise at 1775 cm^− 1^ for both PTzI and CoPTz-DsI, 1780 cm^− 1^ for CoPTz-HaI (symmetric), and 1712–1719 cm^− 1^ (asymmetric). The C–H aliphatic stretch was observed between 2935 and 2849 cm^− 1^ in CoPTz-HaI, which has aliphatic chains in its structure, along with a characteristic C–N stretch peak at around 1399 cm^− 1^. The C-N band for both PTzI and CoPTz-DsI was seen at 1375 cm^− 1^, whereas the C = O bending absorption bands of imide were detected in the range of 728–775 cm^− 1^ for all polyimides. At 3400 cm^− 1^, the carboxylic acid groups in PAA are visible due to the O-H stretching bonds, but the weak amine base may deprotonate free carboxylic acid groups^39–41^. The formation of the imide rings and the usual completion of the imidization process are already visible in the FT-IR spectra of PIs.

### The physicochemical characterization of PIs

At room temperature, the solubility of PTzI, CoPTz-DsI, and CoPTz-HaI was evaluated in a variety of organic solvents (both polar and nonpolar) and hydrocarbon fuels at a concentration of 0.2 weight%. In a test tube with a suitable stopper, add 1.0 mg of polymer sample to 5 mL of each solvent. After a good shaking, cover the test tubes and let them sit for 24 h. The findings in Table 1 showed that the PIs were resistant to the concentrated H_2_SO_4_, and they were insoluble when exposed to toluene, ethanol, gasoline, benzene 95, DMAc, DMF, DMSO, crude oil, and industrial water. Only CoPTz-HaI showed partial solubility in kerosene.

**Table 1:** Solubility behaviour of PIs in various solvents.

Solvent	PTzI	CoPTz-DsI	CoPTz-HaI
Ethanol	**--**	**--**	**--**
DMF	**--**	**--**	**--**
DMAc	**--**	**--**	**--**
DMSO	**--**	**--**	**--**
Toluene	**--**	**--**	**--**
Industrial water	**--**	**--**	**--**
Kerosene	--	**--**	**+-**
Crude Oil	--	**--**	**--**
Gasoline	--	**--**	**--**
Benzene 95	--	**--**	**--**
H_2_SO_4_ 60%	--	**--**	**--**
H_2_SO_4_ 80%	--	**--**	**--**

++ soluble, -+ partially soluble, -- insoluble at room temperature.

In contrast, the PAA precursors, which were formed and soluble in polar aprotic solvents such as DMAc, DMF, and DMSO, while the imidized polymers exhibited insoluble behavior. The reduction in solubility upon thermal imidization is consistent with the conversion from flexible hydrogen-bonded PAA structures to rigid aromatic PI networks. The viscosity or molecular weight of completely imidized polyimides could not be determined directly owing to their insolubility in organic solvents. Instead, η_inh_ inherent viscosities of the soluble PAA intermediates in DMF were measured with a concentration of 0.5 g/100 mL DMF solution at room temperature. The observed viscosity values are ( 0.95 dL/g for PTzI, 0.86 dL/g for CoPTz-DsI, and 0.79 dL/g for CoPTz-HaI), supporting the formation of high-molecular-weight polyimides upon thermal cyclodehydration.

FT-IR spectra confirmed this transformation by the disappearance of the broad O–H/N–H stretching band (3400 cm⁻¹) and the emergence of characteristic imide absorptions: asymmetric C = O stretching (1775–1780 cm⁻¹), symmetric C = O stretching (1712–1719 cm⁻¹), and C–N stretching (1399 cm⁻¹). The decreased solubility of the polyimides is attributed to dense molecular packing, enhanced inter- and intramolecular interactions, and possible partial crosslinking formed during thermal imidization^9^.

**Fig. 4:**
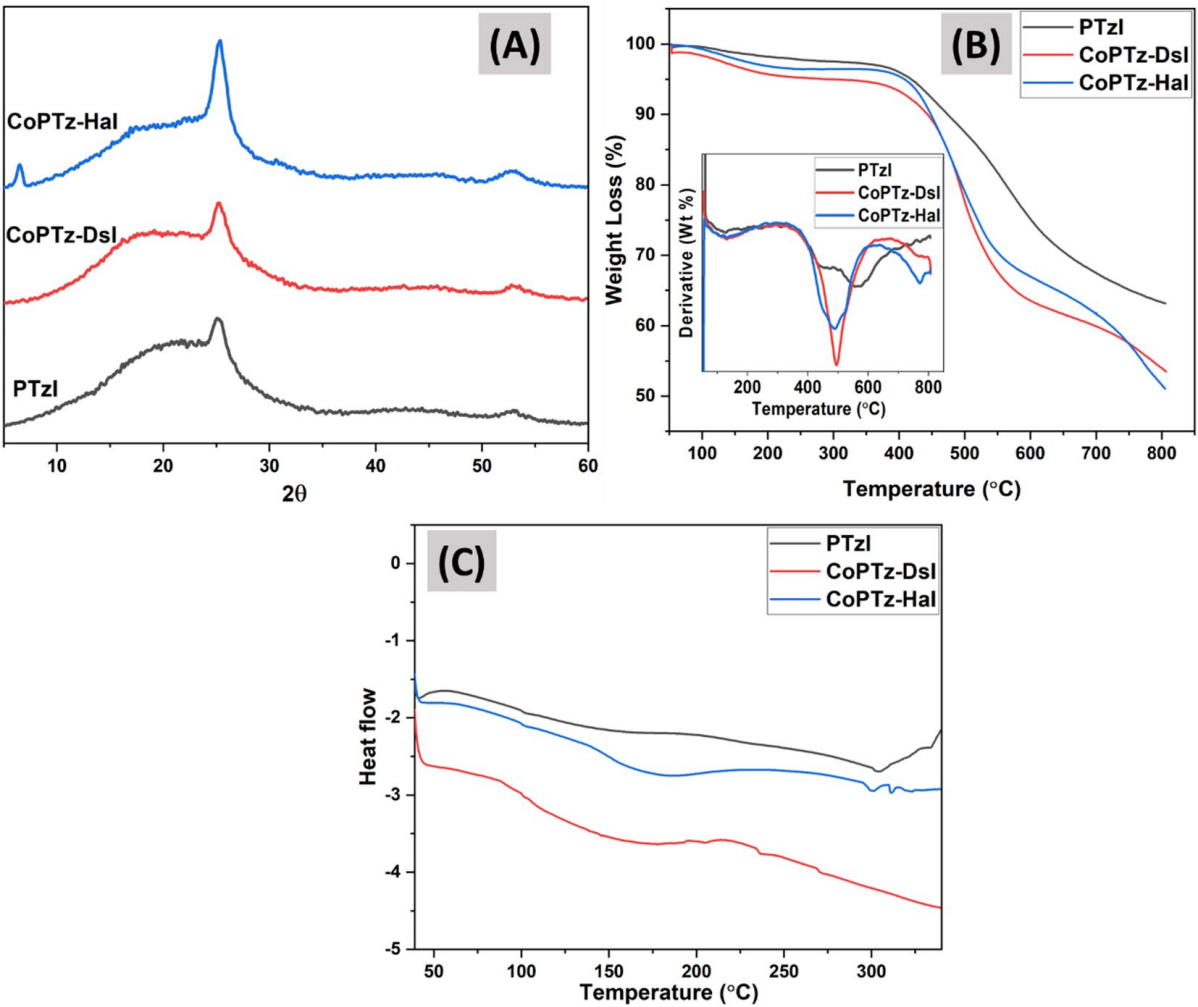
(**A**) XRD patterns, (**B**) TGA thermograms, inset graph (DTG), (**C**) DSC thermograms of PTzI, CoPTz-DsI, and CoPTz-HaI after curing at 300 °C.

The two-dimensional crystalline structure of the PIs samples was examined using a wide-angle X-ray diffractometer with a scanning angle 2θ ranging from 5 to 60 degrees. According to Fig. 4A, the X-ray patterns of polymers exhibit both a broadened area that is disordered at the molecular level, with a narrower characteristic peak at 25 ° that is connected with. This means that polymers may have either a crystalline or an amorphous structure. The X-ray diffractogram area under the curve is used to determine the percentage of crystalline structure in these polymers. PTzI and CoPTz-DsI both have a crystalline percentage of around 13%; however, the crystalline percentage in CoPTz-HaI is approximately 20%. This suggests that all PIs are amorphous, except for CoPTz-HaI, which has a more crystalline structure. The inclusion of aromatic rings in addition to thiazine rings was largely responsible for the production of this amorphous structure. These rings caused the polymer skeleton to become deformed and the orderliness of the polymer chains to become less pronounced. In contrast to other samples, CoPTz-HaI, which is composed of aliphatic chains that include repeated hexamethylene groups, played a role in the buildup of polymer chains that resulted in a more flexible structure^35, 42, 43^.

Table 2; Fig. 4B provide the results and thermograms on the thermal stabilities of the PIs, which were determined using TGA analysis. Derivative thermogravimetric (DTG) analysis reveals that all PI samples exhibit weight loss from the inception of heating to about 217 °C, which might be attributed to residual solvents and residues of PAA. The weight loss (Wt) is minimal, with PTzI losing around 2%, CoPTz-HaI losing about 3.5%, and CoPTz-DsI losing about 4.5%. The initial 5% weight loss temperatures for PTzI and CoPTz-HaI are found to be around 418 °C and 408 °C, respectively, but the temperature for CoPTz-DsI is 294 °C. This finding demonstrates that PTzI and CoPTz-HaI are thermally stable up to 400 °C, in contrast to CoPTz-DsI, which experienced a weight loss of around 5% at a lower temperature. This becomes evident in the subsequent degradation phases, where the stability of these two PIs exceeds that of CoPTz-DsI. The PTzI degrades in a single step, but the other two copolymers degrade in two steps. The PTzI exhibits a main degradation stage that begins at 473 °C, where it loses weight gradually, and retains the most weight around 800 °C, indicating it is quite thermally stable. Among the samples, CoPTz-DsI exhibits the lowest thermal stability, as evidenced by the earliest onset of breakdown and the fastest weight loss, as indicated by its sharp Dr TGA peak at approximately 450 °C.

**Table 2:** Heat rate in (°C) at different decomposition levels in N_2_ at a rate of 10 °C/ min.

PI Sample	T_5%_ (℃)	T_10%_ (℃)	T_20%_ (℃)	T_30%_ (℃)	T_40%_ (℃)	Char Residue (%) at 800 ℃
**PTzI**	**418**	**473**	**563**	**658**	**> 800**	**63**
**CoPTz-DsI**	**294**	**443**	**492**	**534**	**697**	**54**
CoPTz-HaI	408	448	495	554	723	52

Significant values are in bold.

On the other hand, CoPTz-Hal demonstrates slightly better thermal behavior than CoPTz-DsI, with a wider DTG profile and delayed degradation. The stability, however, is still lower than that of the unaltered PTzI. As an initial step in the breakdown of copolymers, cyclization occurs, with the separation of certain end and side groups from the main chain to generate hydrocarbons, water, CO, and COOH. In the second step, the polyimide’s main chain degrades thermally, yielding ammonia gas, CO, and CO_2_. In contrast to CO, which originates directly from the imide ring, the pathway to CO_2_ loss is more complicated. This difficult process might be caused by unreacted anhydrides, PAAs (which can result from incomplete imidization or hydrolysis of imide rings), or the breakdown of isoimides (which can be formed by imide rearrangement)^44^. The degradation behavior of the three copolymers shows that they vary structurally; one polymer, PTzI, has a homogeneous structure and goes through one step of degradation, while the other two show two phases, showing a difference in structure with two structural units^45,46^.

The structural rigidity and decreased solubility of the produced PIs via thermal imidization were further validated by differential scanning calorimetry (DSC). Figure 4C of the DSC traces indicates a low-temperature endothermic feature (50–80 °C) corresponding to residual solvent or moisture, followed by a broad heat-capacity shift associated with the glass transition, as compared to distinct, narrow glass transitions^47^. This behavior is characteristic of rigid aromatic polyimides, resulting from limited mobility and heterogeneous packing. The glass transition temperature (T_g_) of PTzI was 303 °C, CoPTz-HaI was 311 °C, and CoPTz-DsI was 271 °C. The existence of rigid aromatic moieties, which limit chain mobility and decrease solubility, is consistent with these unusually high T_g_ values. A lower T_g_ in CoPTz-DsI indicates less dense chain packing, whereas a higher T_g_ in CoPTz-HaI indicates improved chain packing and intermolecular interactions due to the aliphatic segments. PIs formed by thermal imidization are both structurally rigid and thermally stable, as shown by the DSC data^39,48^.

**Fig. 5:**
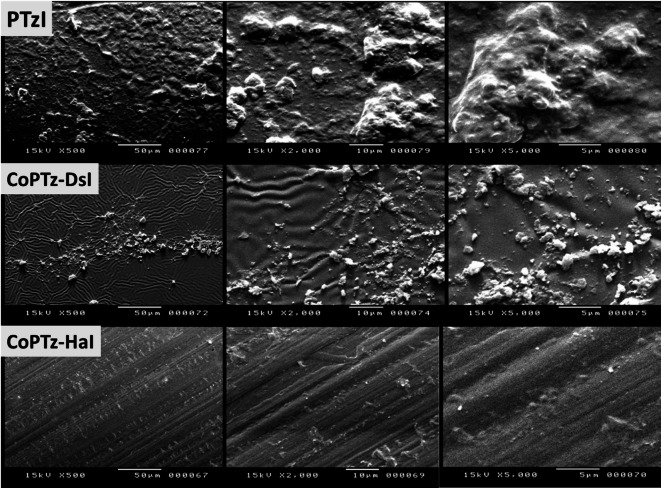
SEM images of PtzI, CoPTz-DsI, and CoPTz-HaI at different magnifications.

As shown in Fig. 5, the morphology of the PI materials was examined using the SEM technique at various magnifications. The surface topography of the produced polymers exhibits structural variations when analyzed using the SEM approach. The surface of the first one, PTzI, is rough and has an uneven distribution of roughness, making it seem like agglomeration or fine grains. The surface of the second one, CoPTz-DsI, is wave-like, ridged, and smoother with tiny wrinkles. The surface of CoPTz-HaI is compact and continuous, with grooves running in both directions and containing tiny particles. The packing, the interactions between chains, and the enhanced arrangements of chains might all be responsible for these variations^38,49,50^. Among all samples, CoPTz-HaI had the highest levels of homogeneity and compactness, leading to a smooth surface and perfectly aligned chains. Its greater (Tg = 311 °C) in comparison to PTzI (303 °C) and CoPTz-DsI (271 °C), as found by DSC, is consistent with this partial ordering. The XRD patterns support these findings; the profile of CoPTz-HaI showed sharper characteristics, suggesting partial ordering and chain alignment. The reason for this is that the existence of the aliphatic chain results in a certain degree of ordering and crystallinity being imparted.

### Electrochemical methods

In this work, the steady state potential (E_s.s_) was determined using potentiodynamic methods. This value is near the corrosion potential (E_corr_ = E_ocp_). The E_im_ was measured on an unplated MS and with various inhibitor samples. One relevant property that potentiodynamic polarization (PP) may record is the corrosion rate (C.R mm/year mpy), the efficiency of inhibition (IE%), surface coverage (θ = IE%/100), corrosion potential (E_corr_ mV), and corrosion current density (I_corr_ µA/cm²). A reference electrode (SEC) and a working electrode are required by standard operating procedures (SOPs), however, the PP studies made use of an additional secondary electrode (Pt wire). All measurements were conducted using the reference voltage that the SEC supplied. Synthetic PI and copolyimides-coated MS (PTzI, PTz-DsI, and PTz-HaI) or uncoated blank samples were used as the working electrodes. The passage of current was enabled by the secondary electrode, which completed the electrochemical circuit. An EG&G Model 273 A potentiostat/galvanostat was used to measure the OCP and PP curves. E_corr_ and TF had a voltage differential of ± 250 mV, and the rate of scan for the change of factor was 0.3 mV/Sec. Following the determination of I_corr_, the tested inhibitors’ CR and IE% were quantitatively ascertained by using Eqs. 1 and 2. This correlation between CR (mpy) and corrosion current density (µA/cm²) is shown by this equation, and it illustrates the occurrence of the corrosion process^34,51–53^.

1 $$\:\text{C}\text{R}=\:\frac{0.13\times\:\text{I}\text{c}\text{o}\text{r}\text{r}\times\:\text{E}\text{q}.\text{W}\text{t}}{{\uprho\:}\times\:\text{A}}$$

2 $$\:\text{I}\text{E}\text{\%}=\frac{\text{C}\text{R}-\text{C}\text{R}1}{\text{C}\text{R}}\:\times\:100\:$$

Equation (1) A stands for the submerged area in cm² in the tested solution, and Eq.Wt is the equivalent weight in gm/eq is equivalent to 55.8 atomic weight. Additionally, the density, denoted as ρ, is 7.874 g/cm³. The time unit is converted to units of measurement with a factor of 0.13. Both the inhibitory and non-inhibitory corrosion rates are denoted by CR and CR1, respectively in Eq. (2).

### Evaluation of anticorrosion characteristics by electrochemical testing

Although polyimides are generally recognized for their corrosion resistance and inhibitory behavior, determining corrosion parameters is essential to quantify and validate their performance. Electrochemical measurements such as polarization curves and impedance spectroscopy provide numerical values (I_corr_, E_corr_, IE%), which allow (i) assessing the efficiency of inhibition, (ii) identifying the inhibition mechanism (anodic, cathodic, or mixed), and (iii) comparing the protective performance of different polyimide structures. Without these parameters, the inhibitory action of PIs cannot be rigorously evaluated or benchmarked for practical applications.

### Evaluating the open circuit potential (E_OCP_)

**Fig. 6:**
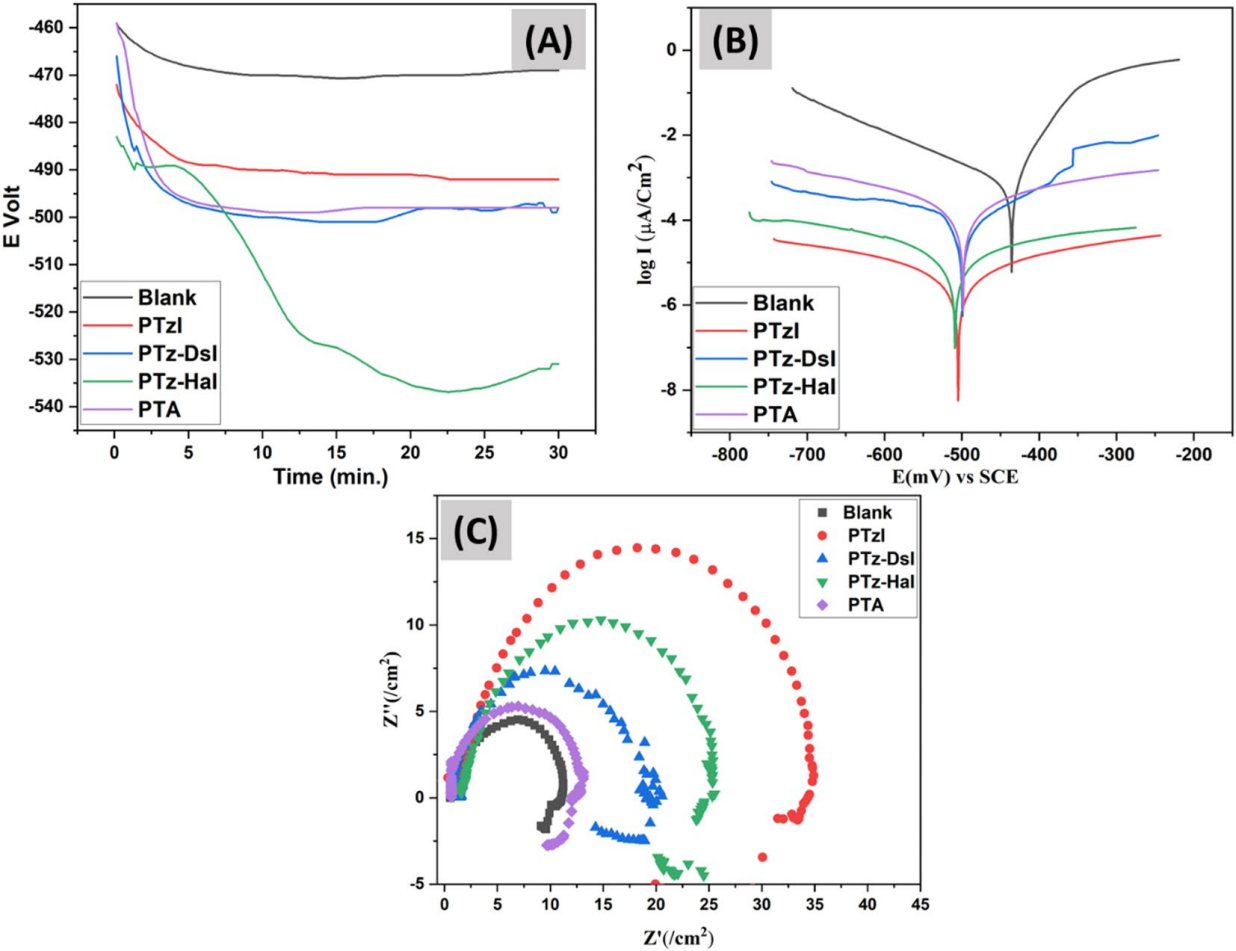
(**A**) E_ocp_–time plots. (**B**) Tafel plots. (**C**) Nyquist plot uncoated MS and MS coated with PTzI, PTz-DsI, PTz-HaI, and PTA.

The first stage of doing an electrochemical corrosion investigation is to ascertain the open circuit potential OCP. The OCP is a cornerstone for corrosion characterization in this context, as it is related to the system’s corrosion potential. Another factor that contributes to OCP during corrosion is the electrode’s corrosion resistance in a certain electrolyte. To establish the voltage of the electrochemical cell and demonstrate the correlation between time (t) and voltage (E) in the absence of the counter electrode, the OCP approach is used. A higher OCP number indicates a greater corrosion resistance. The steady-state voltages (E_s.s_) of uncoated blank MS specimens submerged in a 1.0 M H_2_SO_4_ solution and samples coated with PI and co-polyimides are shown in Fig. 6A; Table 3. There is a substantial change in the blank sample’s steady-state voltage (E_s.s_) when contrasted with its immersion voltage (E_im_). This change is caused by the prepared polymers adhering to the active surface areas and being exposed to oxidation, resulting in the coating of the MS electrode’s surface with a thin oxide layer to protect the MS from corrosion.

**Table 3:** Potential (mV) against time (min.) and potentiodynamic polarization parameters of MS immersed in 1.0 M H_2_SO_4_ with PTzI, PTz-DsI, PTz-HaI, and PTA.

Inhibitors	-E_im_	-E_s.s_	I_corr_ (µA/Cm^2^)	C.*R*	IE%	θ	*R*_ct_ Ω.cm^2^
1.0M H_2_SO_4_	**459**	**469**	**2699**	**2488.57**	**-----**	**-----**	1.283E + 01
PTzI	**472**	**492**	**19.18**	**17.68**	**99.35**	**0.99**	3.336E + 01
PTz-DsI	**466**	**498**	**141.3**	**130.28**	**95.17**	**0.95**	2.448E + 01
PTz-HaI	**531**	**483**	**104.5**	**96.35**	**96.43**	**0.96**	1.892E + 01
PTA	459	498	613.4	565.58	79.05	0.79	1.328E + 01

Significant values are in bold.

### **Evaluating Tafel polarization and electrochemical impedance spectroscopy (EIS)**

To assess the coatings’ corrosion protection capabilities and learn how structural alteration improved their properties, electrochemical experiments including (EIS) and Tafel polarization were performed. On applying (EIS) and Tafel measurements, the corrosion resistance behavior of PTA monomer, pristine PI, and co-polyimide coatings produced in a 1.0 M H₂SO₄ solution was examined. Table 3 details the electrochemical parameters derived via Tafel extrapolation, including the CR, corrosion potential (E_corr_), and corrosion current density (I_corr_). Figure 6B shows the Tafel graphs of PTA, PTzI, PTz-DsI, and PTz-HaI. Corrosion behavioral patterns are often low when corrosion current densities are low and corrosion potentials are positive. The addition of co-polyimide derivatives and PI was shown to alter the Tafel slope. Placing inhibitor compounds onto the MS electrode surface was the first step, leading to a positive difference between the blank solution and the inhibitors’ E_corr_ not exceeding 85 mV. The resulting decrease in the anodic and cathodic Tafel slopes suggested that the inhibitors were mixed types^54^. When the inhibitors under study were not present, I_corr_ rose to 2699 (µA cm^− 2^) and CR was 2488 mpy; nevertheless, samples coated with inhibitors exhibited higher corrosion resistance than bare MS. The control solution’s I_corr_ and CR values were decreased and the IE% was enhanced when inhibitors were added. In comparison to PTz-HaI (104.5 µA cm^− 2^), PTz-DsI (141.3 µA cm^− 2^), and PTA (613.4 µA cm^− 2^), PTzI exhibits the best corrosion inhibition capability, with an I_corr_ value of 19.2 µA cm^− 2^. A corrosion rate (CR) of 17.7 mpy was recorded for PTzI, indicating an extremely high corrosion resistance. This rate is lower than that of without coating (2488.57 mpy), PTz-HaI (96.4 mpy), PTz-DsI (130.3 mpy), and PTA (565.6 mpy). PTzI achieved a greater inhibition efficiency (IE%) of 99.4% compared to PTz-HaI (96.4%), PTz-DsI (95.2%), and PTA (79.1%). One possible explanation for PTzI’s strong inhibitory impact might be the presence of PTA units in the PI structure. This could be because of the structure’s greater number of electrons as a result of the large number of hetero atoms with non-bonding electron pairs. Also, the increase in the amount of benzene rings containing π electrons is responsible for the enhanced inhibitory efficacy of PTzI. Since nitrogen and sulfur atoms interact directly with metal surfaces, their presence improves the corrosion resistance of PTzI coatings^24,25,55^. Variations in the form of the repeating units cause structural differences among these polymers. The adherence to the surface and coverage area are both improved when the number of hetero atoms possessing lone pairs and benzene rings in the amorphous form of PTzI increases^56,57^. On the other hand, the inhibition efficiency was impacted by the fact that PTz-HaI and PTz-DsI, which contain various repeating units like alkane chains and DDS, reduced the amount of PTA monomer in the polymer’s main chain^54,58,59^.

An essential analytical tool in corrosion research is the Nyquist impedance profile (EIS) data presentation. This profile gives useful information on surface characteristics and reaction behavior. The Nyquist curve for MS in 1.0 M H_2_SO_4_ and the charge transfer resistance (*R*_ct_) values for MS with (PTzI, PTz-DsI, PTz-HaI, PTA) and without coating are shown in Fig. 6C; Table 3. In the high-frequency area of the Nyquist plot, a semicircle represents (*R*_ct_) at the metal/coating contact. If the semicircle is bigger, it means the (*R*_ct_) is greater, which means the coating is more resistant to corrosion and has a greater capacity to resist electron transfer. The increase in R_ct_ values is caused by the formation of an insulating protective covering at the metal/solution interface^60^. When comparing the coated samples (*R*_ct_) to the bare MS, the coated samples (PTzI, PTz-DsI, PTz-HaI, PTA) demonstrate better barrier properties for inhibiting corrosion (3.3, 2.4, 1.9, and 1.3 E + 01 Ω.cm^2^, respectively). This is correlated with higher corrosion resistance, as evidenced by the wider semicircle. A variety of hydrophobicity and molecular design factors account for the fact that PIs have varying effects on corrosion inhibition. The broadest semicircle seen in the PTzI sample is indicative of its outstanding barrier qualities and maximum inhibitory effect (99.4%). The incorporation of PTA into the polymer backbone, which raises the electron density and incorporates heterocyclic structures, leads to enhanced performance. These groups improve the adherence of the coating to the steel surface, and the protective characteristics of the coating are also enhanced^61,62^.

### Characteristics of adhesion

**Fig. 7:**
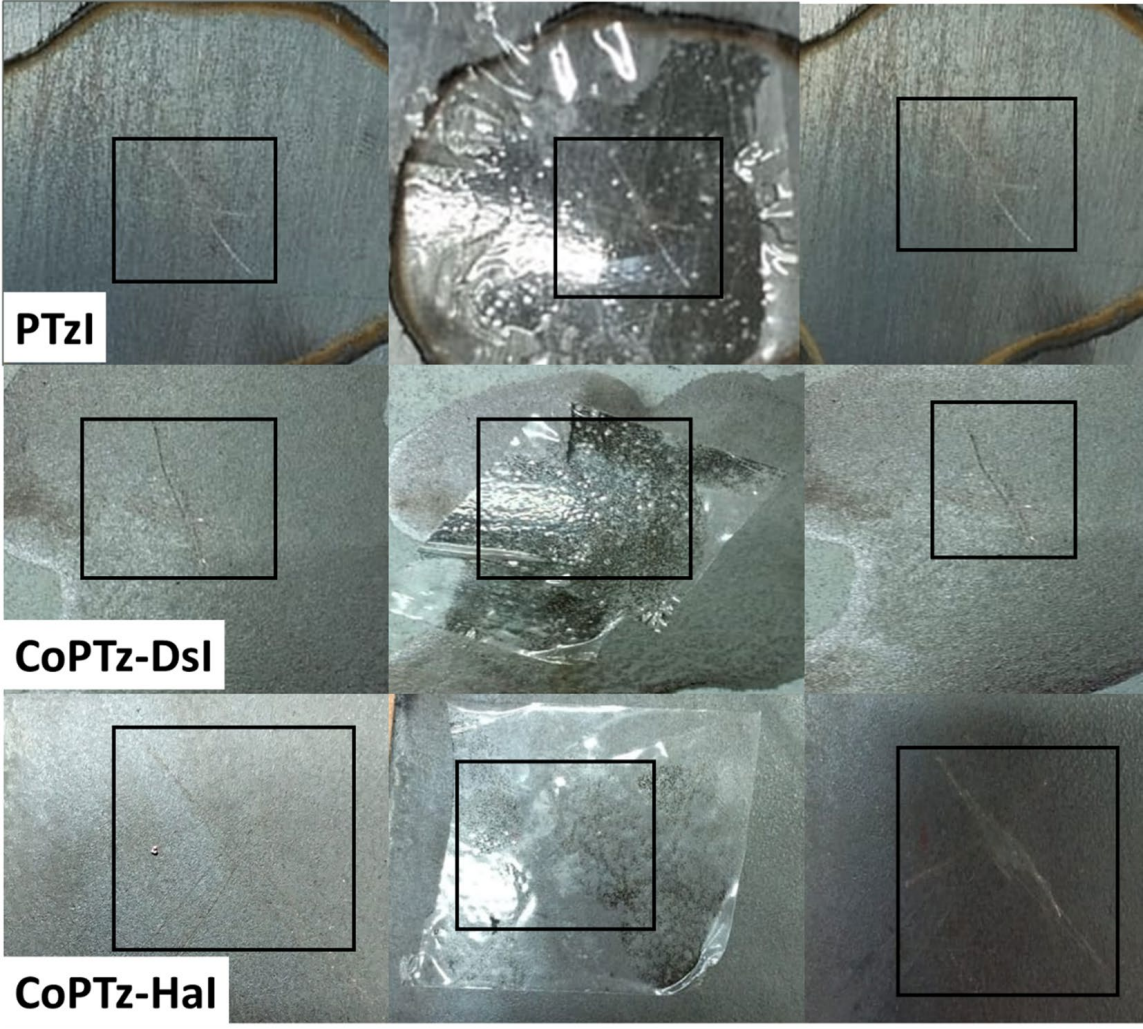
Images of PTzI, CoPTZ-DsI, and CoPTz-HaI for adhesion test according to Standard Test Method D3359 test method A—an X-cut.

In accordance with ASTM D3359 (Standard Test Method for Measuring Adhesion by Tape Test), the adhesion of the PIs was evaluated. The PI samples were gradually cast onto aluminum plates (Fig. 7) and cured in an oven at 300 °C for 24 h. The resulting films were approximately 40 μm thick. After cooling to room temperature, the plates were placed on a stable surface, and an appropriate area was selected under a lighted magnifier. Each film was cut in a single, continuous motion to form an X cut, ensuring the incision reached the steel substrate. The cut surface was gently brushed, after which a segment of adhesive tape was applied over the X-cut and firmly pressed to ensure uniform contact. The tape was then removed rapidly, about 90 s after application, at an angle close to 180°, and the surface was inspected under a magnifier to assess any coating removal^52,63,64^.

According to ASTM D3359 classification, PTzI and CoPTz-DsI exhibited an adhesion rating of 4 A, indicating strong adhesion with minimal detachment at the cut edges. In contrast, CoPTz-HaI showed a rating of 2 A, reflecting partial adhesion with noticeable coating removal along the incision.

## Conclusion

The synthesis of PTzI and its copolymers, CoPTz-DsI and CoPTz-HaI, using the conventional thermal imidization method, illustrates the significant potential of incorporating 1,3-thiazine-based structures into polyimide materials, resulting in improved thermal stability and advantageous functional characteristics. They exhibit the typical characteristics of high-performance polymers owing to their rigid aromatic backbones and imide linkages, which impart chemical resistance and excellent thermal endurance. The high-performance nature was confirmed by remarkable thermal stability, as evidenced by TGA results that showed decomposition temperatures above 440 °C with a significant char residue. Various degrees of surface compactness were reflected in the amorphous shape, which was determined through SEM structural analyses. Outstanding corrosion inhibition properties in acidic conditions were revealed by the polymers, with PTzI having a remarkable protection efficacy of 99.4%. Incorporating the PTA monomeric units into the polymer chain enhanced the materials’ protective qualities, suggesting a potential way to make coatings that are resistant to corrosion via controls over structural modification and content percentage.

## Supplementary Information

Below is the link to the electronic supplementary material.

Supplementary Material 1

## Data Availability

All data generated or analyzed during this study are included in this published article [and its supporting information files].
