# Supplementary material for: Design and thermal imidization of new 1,3-thiazine-based polyimides and copolyimides for high-performance corrosion inhibition
Source: Sci Rep. 2025 Oct 27;15:37354. doi: 10.1038/s41598-025-22235-4 (PMC12559712; doi:10.1038/s41598-025-22235-4)
Supplement: Supplementary file 1 — Supplementary Material 1 [file 41598_2025_22235_MOESM1_ESM.docx]

**Supporting Information**

**Design and Thermal Imidization of New 1,3-Thiazine-Based Polyimides and Copolyimides for High-Performance Corrosion Inhibition**

Marwa M. Sayed^1, *^, Shimaa M. Ahmed^2^, Mohamed Abdel-Hakim^3^, El-Refaie Kenawy^4^ and Kamal I. Aly^2, *^

^1^ Chemistry Department, Faculty of Science, New Valley University, El-Kharja 72511, Egypt.

^2^ Polymer Research Laboratory 122, Chemistry Department, Faculty of Science, Assiut University, 71516, Egypt.

^3^Chemistry Department, Faculty of Science, Al-Azhar University, Assiut 71524, Egypt.

^4^Polymer Research Group, Chemistry Department, Faculty of Science, Tanta University,

Tanta, 31527, Egypt.

**Corresponding Authors:**

Kamal I. Aly Email: [Kamalaly@aun.edu.eg](mailto:Kamalaly@aun.edu.eg)

Marwa M. Sayed Email: marwa.m@sci.nvu.edu.eg


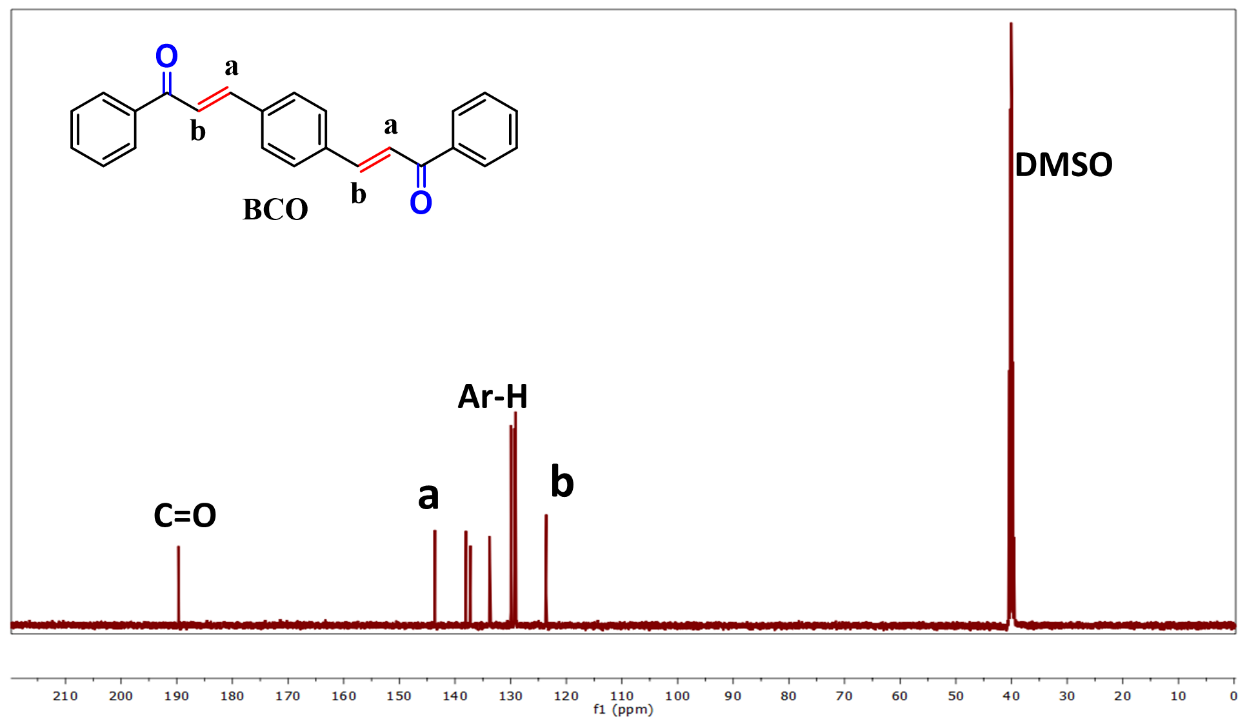

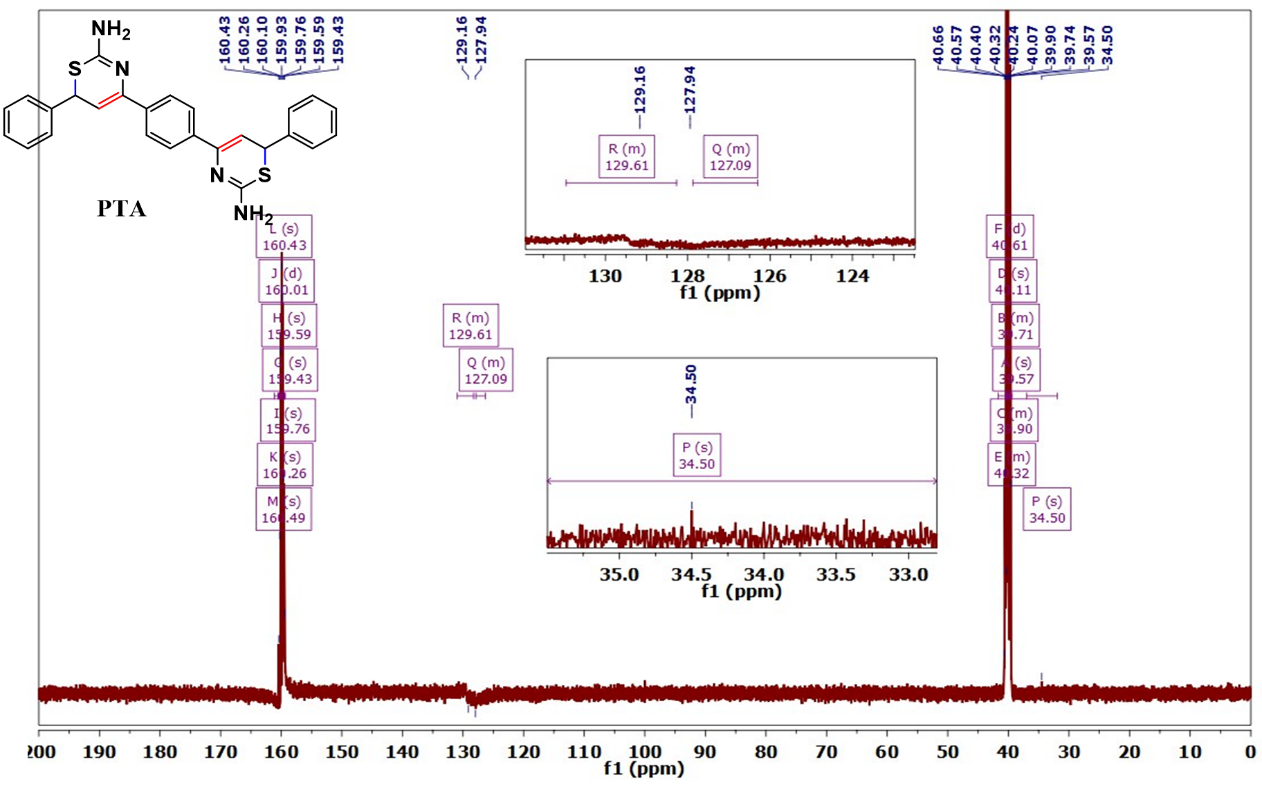


**Figure S1.** ^13^C-NMR of BOC and PTA compounds.


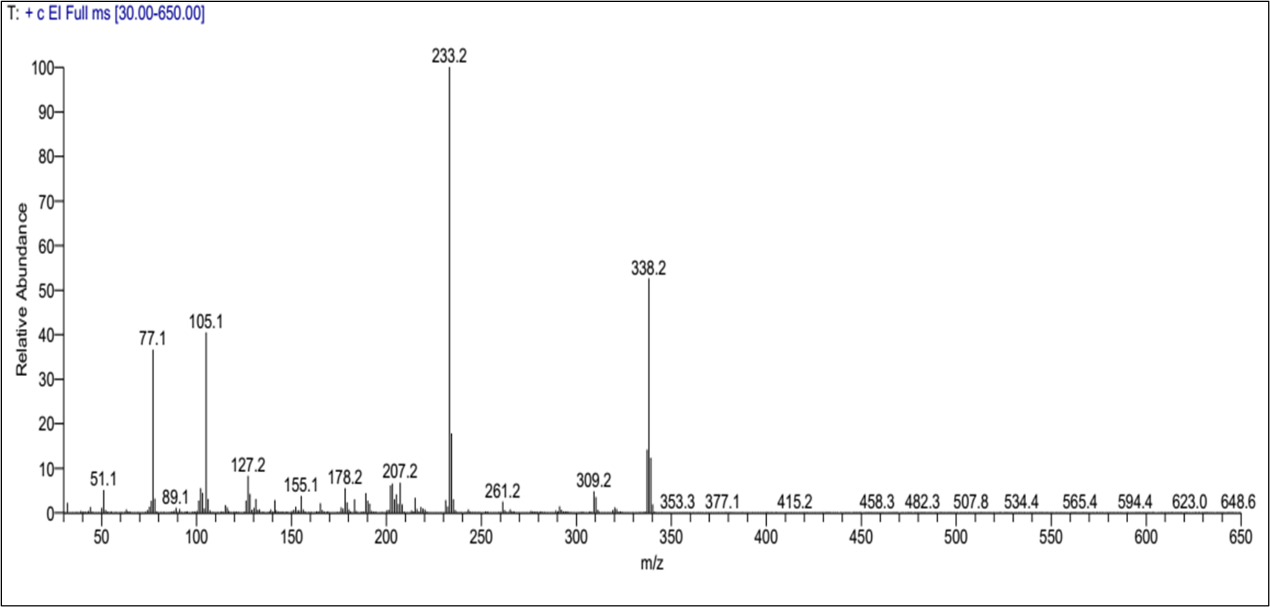


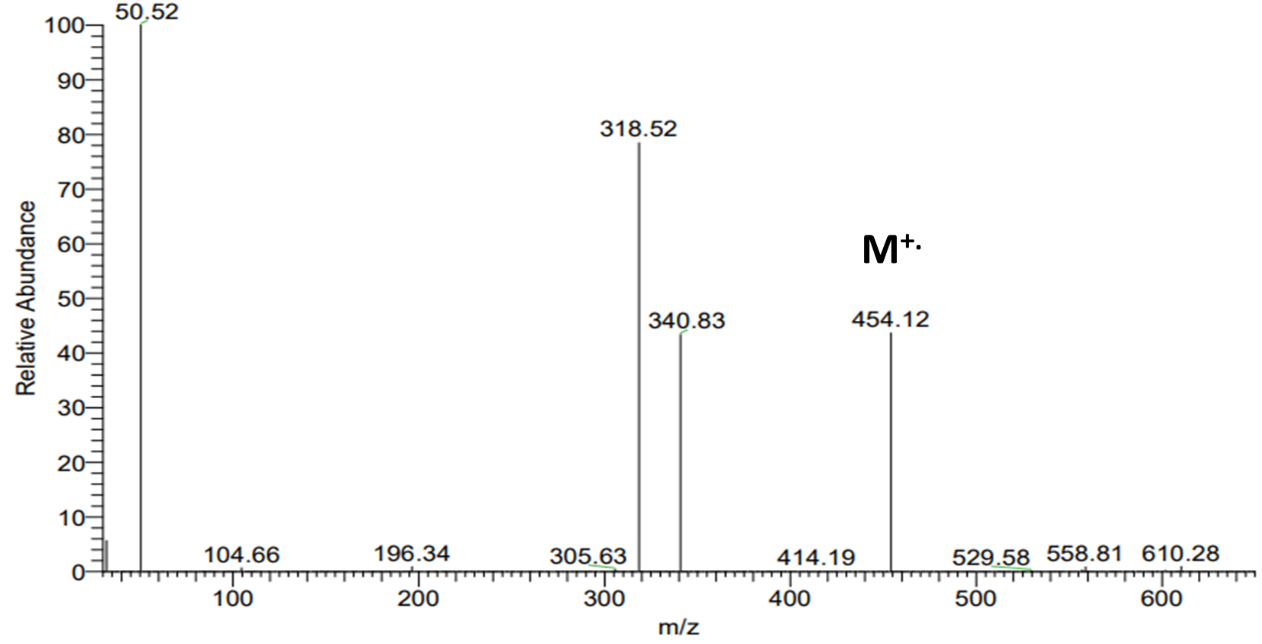


**Figure S2.** Mass spectrum of BOC and PTA compounds.
